# The ability to utilise ammonia as nitrogen source is cell type specific and intricately linked to GDH, AMPK and mTORC1

**DOI:** 10.1038/s41598-018-37509-3

**Published:** 2019-02-06

**Authors:** Shervi Lie, Tingting Wang, Briony Forbes, Christopher G. Proud, Janni Petersen

**Affiliations:** 1 Flinders Centre for Innovation in Cancer, College of Medicine and Public health, Flinders University, Adelaide, SA 5042 Australia; 2 Nutrition and Metabolism, South Australia Health and Medical Research Institute, North Terrace, PO Box 11060, Adelaide, SA 5000 Australia

## Abstract

Ammonia can be utilised as an alternative nitrogen source to glutamine to support cell proliferation. However, the underlying molecular mechanisms and whether all cells have this ability is not fully understood. We find that eleven cancer and non-cancerous cell lines have opposite abilities to tolerate and utilise ammonia to support proliferation in a glutamine-depleted environment. HEK293, Huh7, T47D and MCF7 cells can use ammonia, when starved of glutamine, to support proliferation to varying degrees. Glutamine depletion reduced mTORC1 activity, while additional ammonia supplementation diminished this mTORC1 inhibition. Depletion of glutamine promoted a rapid and transient activation of AMPK, whereas, additional ammonia supplementation blocked this starvation-induced AMPK activation. As expected, drug-induced AMPK activation reduced cell proliferation in glutamine-depleted cells supplemented with ammonia. Surprisingly, mTORC1 activity was largely unchanged despite the enhanced AMPK activity, suggesting that AMPK does not inhibit mTORC1 signalling under these conditions. Finally, glutamate dehydrogenase (GDH) inhibition, a key enzyme regulating ammonia assimilation, leads to AMPK activation, mTORC1 inhibition and reduced proliferation. Ammonia provides an alternative nitrogen source that aids certain cancer cells ability to thrive in nutrient-deprived environment. The ability of cells to utilise ammonia as a nitrogen source is intricately linked to AMPK, mTORC1 and GDH.

## Introduction

Cell growth and proliferation are highly dependent on nutrient availability. In eukaryotes, target of rapamycin (TOR) signalling network is essential in sensing nutrient abundance and coordinating growth and proliferative signals^1^. In all organisms, TOR forms two structurally and functionally distinct complexes^2^. Mammalian target of rapamycin complex-1 (mTORC1) is defined by its interacting protein, raptor, while mTOR complex-2 (mTORC2) is defined by its interaction with rictor. The rapamycin-sensitive TORC1 is a major nutrient sensor that integrates environmental cues with cell growth and proliferation. Certain amino acids are key activators of TORC1 signalling which in turn stimulates anabolic processes, including protein synthesis, growth and proliferation^3^.

Nitrogen is an essential element for protein and nucleotide synthesis, and is hence needed to support growth and proliferation. A recent report showed that nitrogen sources can activate TORC1 via glutamine synthesis^4^. More importantly, glutamine has been reported to induce nucleotide synthesis and thus support proliferation in glutamine-depleted glioblastoma cells by inducing glutamine synthetase (GS) activity^5^. Ammonia is a common metabolic by-product that can be assimilated into glutamine, and hence acts as an indirect nitrogen source. In mammals, GS and glutamate dehydrogenase (GDH) are the key enzymes required for ammonia assimilation^6^. Expression of GS and GDH is significantly increased in many cancers^7,8^. Recent studies showed that GDH rather than GS is the key enzyme in ammonia assimilation into glutamate, as a precursor to glutamine and more importantly, these reports showed that ammonia can support cell growth in T47D and MCF7 breast cancer cell lines^7,9^. These studies support earlier findings by Meng *et al*. which showed that ammonia can act as an alternative nitrogen source and support hepatoma (HEP3B) cell proliferation through its assimilation into glutamate^10^. In support of these findings, ammonia was shown to induce activation of mTORC1 and mTORC2 and to promote MCF7 cell proliferation^11^. This is consistent with our previous finding which showed that ammonia can re-activate mTORC1 signalling in Hep3B cells cultured in a glutamine-depleted environment^12^. Interestingly, however, Spinelli *et al*. reported that fibroblast cells are unable to utilise ammonia to support their growth^7^, suggesting that cells differ in their ability to utilise ammonia as an alternative nitrogen source.

AMP-activated protein kinase (AMPK) is a well-characterised energy sensor that regulates cellular processes in response to environmental cues^13^. AMPK is predominantly regulated by glucose availability and environmental stress. Its role in inhibiting mTORC1 during nutritional challenge is also well established^13^. Although previous studies have provided evidence that ammonia can be used as an alternative nitrogen source to support cell proliferation in a number of cancer cells^7,9–11^, the report that showed fibroblast cells cannot use ammonia to support their growth^7^, opened up a question of whether this ability is unique to cancer cells and whether all cancer cells have this ability. Furthermore, we have shown that AMPK can sense nitrogen stress and thus inhibit mTORC1 in yeast^12^. However, the effects of nitrogen stress and ammonia supplementation in mammalian cells on AMPK are unknown. Therefore, in this study we aimed to screen a panel of cancer and non-cancerous cell lines for their ability to utilise ammonia as an alternative nitrogen source to support proliferation. We determined the effects of glutamine depletion with or without ammonia supplementation on AMPK and mTORC1 activation during acute and chronic exposure, as well as the effects of activating AMPK and inhibiting GDH on mTORC1 activity and cell proliferation.

## Results

### Different cell types have varying abilities in tolerating glutamine depletion and utilising ammonia as an alternative nitrogen source

Ammonia has been considered a toxic by-product that needs to be removed from cells and subsequently cleared in the liver through the urea cycle^6,14^. However, recent reports have provided evidence that ammonia can support cell growth in tumor cells at much higher concentrations than are normally considered toxic to primary human astrocytes (5 mM)^7^. In this and a previous study^12^, 0.8 mM NH_4_Cl was used as an alternative nitrogen source, consistent with other studies which has determined this concentration as optimum and relevant to the tumor microenvironment^7,10^.

**Figure 1:**
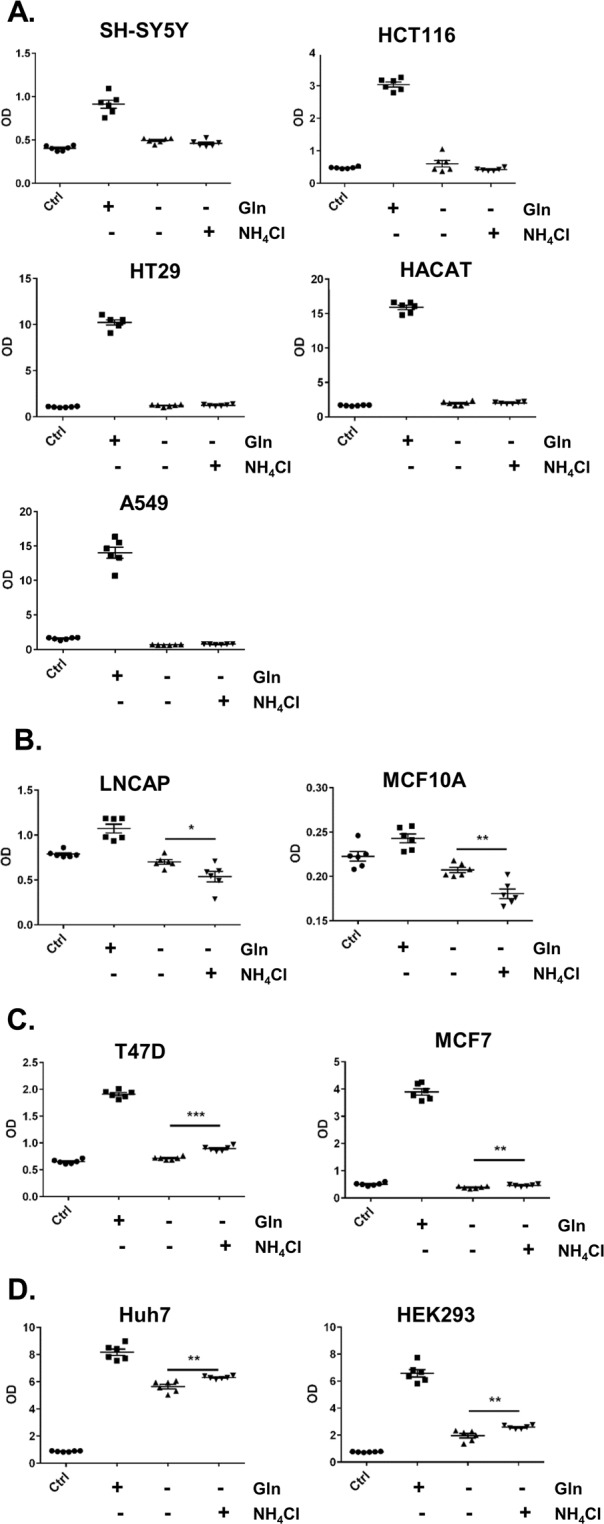
Tolerance of varying cell types of glutamine depletion and ammonia utilisation as an alternative nitrogen source. Cells in the control group were fixed in 4% formaldehyde 24 h after plating (n = 6). This represents the starting number of cells grown in normal media (DMEM containing high glucose, 10% FBS and 8 mM glutamine). Cells in the other groups (n = 6) were washed with DPBS and fresh media with or without glutamine or 0.8 mM NH_4_Cl was added. After 3 days, cells in all groups were fixed and stained with 0.5% crystal violet solution in 4% formaldehyde and colorimetric (OD) measurement was quantified using spectrophotometer. SH-SY5Y, HCT116, HT29, HACAT and A549 cells can survive in glutamine-depleted culture, but cannot utilise NH_4_Cl to proliferate (A). LNCAP and MCF10A cells displayed cell loss in glutamine-depleted media that was worsen by NH_4_Cl supplementation (**B**). MCF7 and T47D cells can survive in glutamine-depleted culture and they can utilise NH_4_Cl as alternative nitrogen source to support proliferation (**C**). Huh7 and HEK293 cells can proliferate in glutamine-depleted culture and proliferation was enhanced in the presence of NH_4_Cl (**D**). *p ≤ 0.05, **p ≤ 0.01, ***p ≤ 0.001.

We performed growth assays over three days and used crystal violet staining to determine the degree of cell proliferation, on a number of cancer (SH-SY5Y, HCT116, HT29, LNCAP, A549, MCF7, T47D and Huh7) and non-cancerous (HACAT, MCF10A and HEK293) cell lines. We found that the neuroblastoma (SH-SY5Y), colorectal carcinoma (HCT116), colorectal adenocarcinoma (HT29) and human lung carcinoma cells (A549) cells cannot utilise NH_4_Cl to support proliferation in glutamine deprived environments (Fig. 1A) as their relative cell numbers were unchanged after 3 days when compared to the control, which represent the number of seeded cells prior to glutamine starvation. In contrast, non-starved glutamine-containing media promoted cell growth (Fig. 1A). The human prostate adenocarcinoma cells, LNCAP, and the human mammary cell line, MCF10A, also cannot utilise NH_4_Cl for proliferation. In contrast, they were sensitive to NH_4_Cl and displayed cell death (Fig. 1B). Consistent with previous reports, the human breast cancer cell lines, MCF7 and T47D^7,9,11^ can survive without glutamine and were able to utilise NH_4_Cl as alternative nitrogen source to support some proliferation in glutamine depleted media (Fig. 1C). Interestingly, in two out of the eleven cell lines we tested: the human hepatocellular carcinoma cells, Huh7, and, to a lesser extent, the human embryonic kidney cells, HEK293, were not only able to utilise NH_4_Cl to proliferate in glutamine-depleted environment, but could also proliferate in glutamine-depleted media without NH_4_Cl supplementation (Fig. 1D).

**Figure 2:**
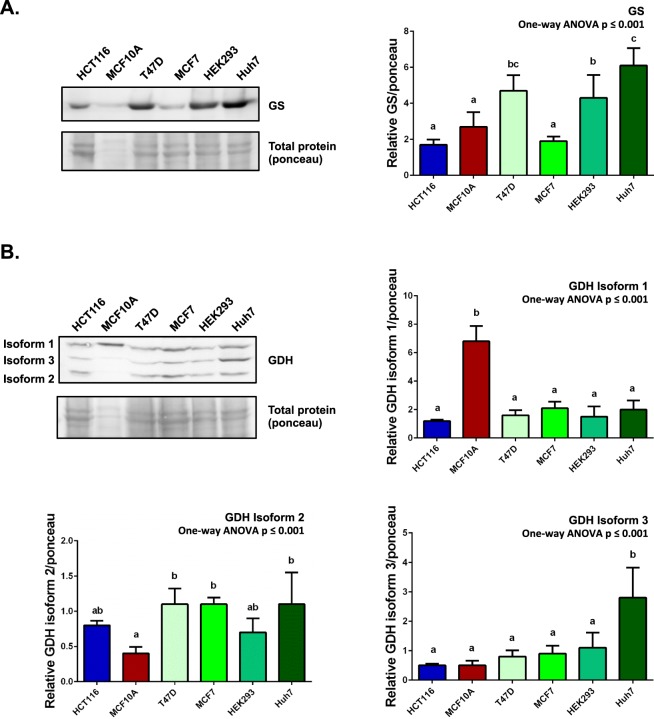
Glutamine synthetase and glutamate dehydrogenase protein abundance varies in different cell lines. Cells (HCT116, MCF10A, T47D, MCF7, HEK293 and Huh7) were plated in complete medium containing glutamine for 24 h. Cultures were lysed and collected for western blotting 24 h later. Levels of glutamine synthetase (GS) (**A**) and glutamate dehydrogenase (GDH) (**B**) relative to total protein (ponceau) were quantified using western blot. Note that MCF10A yielded very low amount of protein (total protein loaded ~4–16x more than other samples). (n = 3) One-way ANOVA p ≤ 0.001 demonstrates significantly different protein levels across samples, letters represent statistically distinct groups. Full-length blots are presented in Supplementary information Fig. 4

GS and GDH enzymes are required for ammonia assimilation^6^ and were shown to be significantly increased in many cancers^7,8^. We next measured the protein level of GS and GDH in HCT116 (cannot utilise ammonia), MCF10A (sensitive to ammonia), T47D, MCF7, HEK293 and Huh7 (can utilise ammonia to proliferate) cells to assess whether their levels of expression correlated with the ability to utilise ammonia (Fig. 2A,B). All cells expressed both GS and 3 isoforms of GDH, albeit at different levels. The levels of GS and GDH’s did not appear to correlate with the sensitivity or ability of these cells to survive or utilise ammonia to support proliferation.

**Figure 3:**
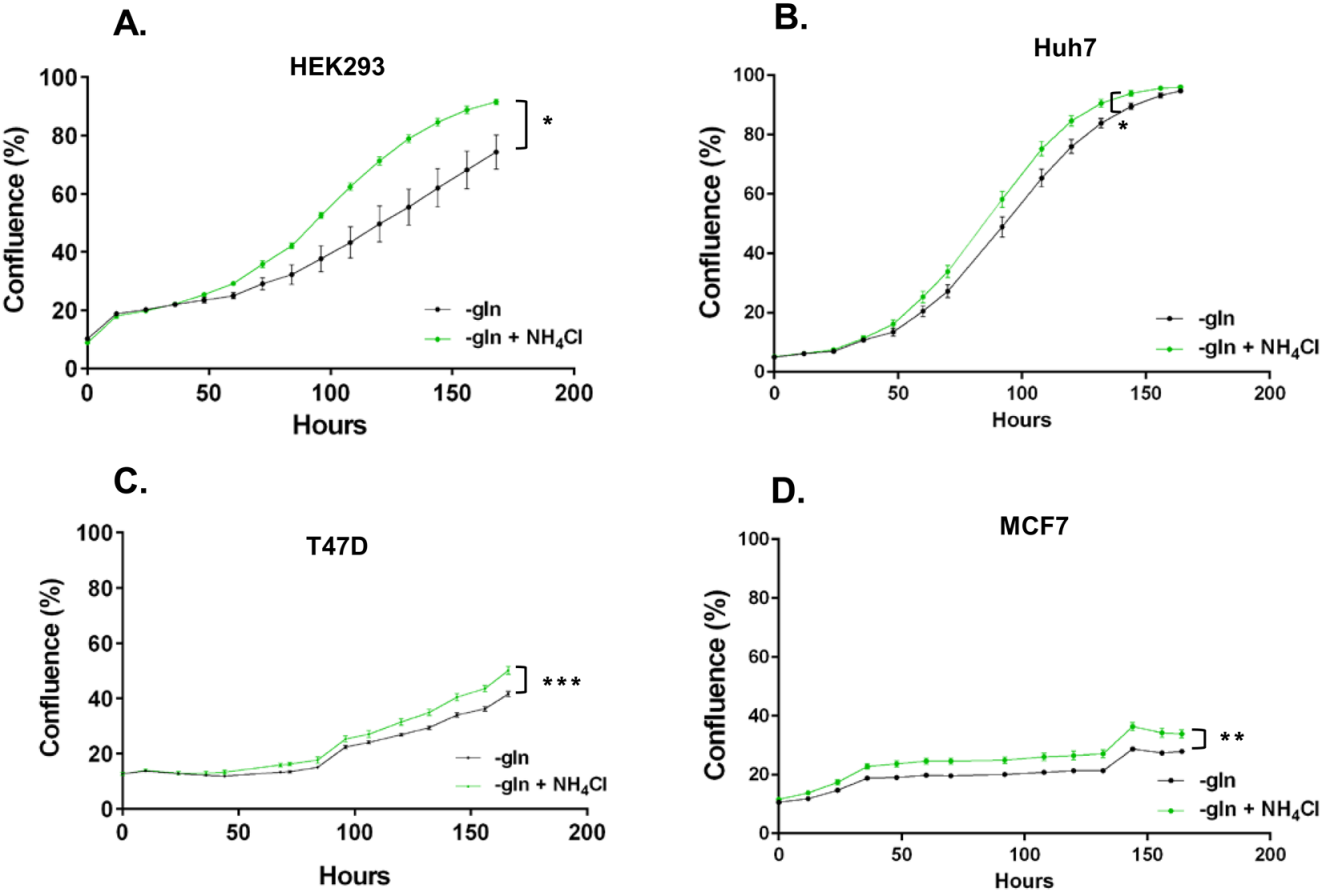
Proliferation in the presence or absence of ammonia in glutamine-depleted medium. HEK293 (**A**), Huh7 (**B**), T47D (**C**) and MCF7 (**D**) cells were plated and 24 h later they were washed with DPBS and cultured in fresh glutamine-depleted medium or glutamine-depleted medium with NH_4_Cl (n = 6). Cell proliferation was then measured based on live imaging using the Incucyte FLR assay over 7 days. *p ≤ 0.05, **p ≤ 0.01, ***p ≤ 0.001.

Next we chose two cell lines that were able to utilise NH_4_Cl as an alternative nitrogen source to proliferate, but were unable to proliferate in glutamine-depleted environment, i.e., T47D and MCF7, and two cell lines that were able to proliferate in glutamine-depleted environment with or without NH_4_Cl, i.e., HEK293 and Huh7, for further studies. We confirmed the growth assay results on these cells using the IncuCyte, which measures cell confluence based on images taken of the live cells over 7 days. In all 4 cell lines ammonia supplementation increased cell proliferation compared to cells cultured in glutamine-depleted media without ammonia (Fig. 3A–D). Interestingly, over 7 days, T47D and MCF7 (to a much lesser extent) cells also showed an ability to grow in glutamine depleted media (Fig. 3C,D)

### Glutamine depletion and ammonia regulate mTORC1 and AMPK signalling

**Figure 4:**
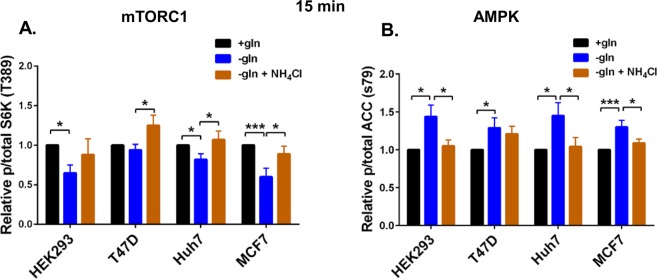
Acute glutamine depletion and ammonia regulate AMPK and mTORC1 signalling. Cells (HEK293, Huh7, T47D and MCF7) were plated in complete medium containing glutamine for 24 h. One culture was lysed and collected for western blotting 24 h later (T0). The remainder of the cultures were washed with DPBS and fresh glutamine-depleted media with or without 0.8 mM NH_4_Cl was added. Cultures were collected and lysed at 15 min for western blotting. Levels of phosphorylated S6K (T389) relative to total S6K protein levels (**A**) and phosphorylated ACC (S79) relative to total ACC protein levels (**B**) were quantified using western blot. *p ≤ 0.05, **p ≤ 0.01, ***p ≤ 0.001.

In our previous study, we showed that hepatocellular carcinoma cells, Hep3B, responded to glutamine depletion with reduced mTORC1 signalling, as reflected by reduced S6K phosphorylation, and that NH_4_Cl treatment restored mTORC1 activity^12^. S6K is a direct substrate of mTORC1 and hence a well-established marker of mTORC1 activity^15^. Here we showed that, consistent with the findings in Hep3B cells, glutamine depletion (−gln) resulted in a decrease in mTORC1 activity in HEK293, Huh7 and MCF7 cells, but not T47D at 15 min post-incubation compared to cells grown in normal media (+gln) (Fig. 4A). At 15 min post-incubation, NH_4_Cl supplementation in glutamine-depleted environment increased mTORC1 signalling compared to glutamine depletion without supplementation in T47D, Huh7 and MCF7 cells (Fig. 4A). Interestingly, in all four cell lines acute (15 min) glutamine depletion (−gln) increased phosphorylation of acetyl-CoA carboxylase (ACC), which is a direct substrate and hence a marker of AMPK activity^16^ when compared to cells cultured in full media (+gln) (Fig. 4B). AMPK is known to be upregulated during nutrient deprivation, and in particular to glucose deprivation^13^. However, this is the first report to show that acute deprivation of a specific amino acid also increases AMPK activation. Supplementation with NH_4_Cl (−gln + NH_4_Cl) in glutamine-depleted culture reduced AMPK activity (at 15 min) when compared to glutamine-starved cells (−gln) in HEK293, Huh7 and MCF7 cells (Fig. 4B). It is well established that AMPK and mTORC1 have opposing effects on each other’s activity^13^. Consistently, these results show that acute glutamine depletion promotes AMPK activity in all 4 cell lines and that additional NH_4_Cl supplementation reduces AMPK and promotes mTORC1 activity in three of the four cell lines.

**Figure 5:**
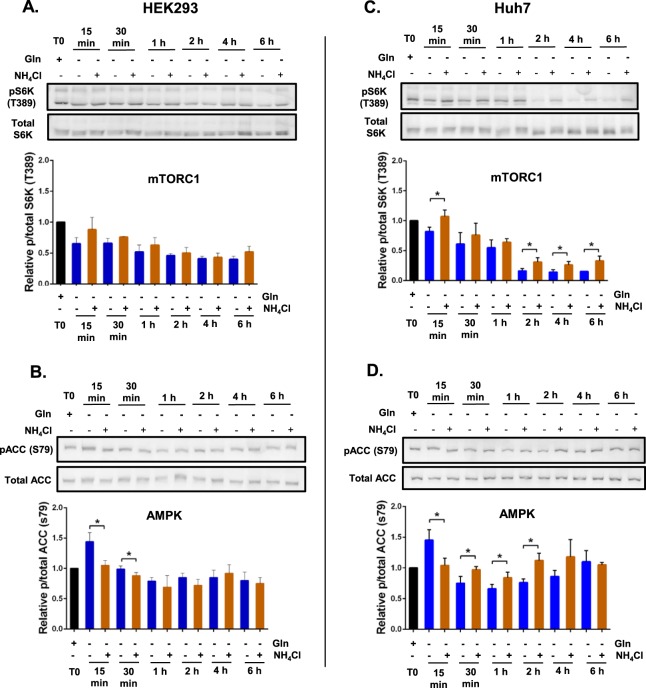
Acute glutamine depletion and ammonia lead to dynamic changes in mTORC1 and AMPK signalling in HEK293 and Huh7 cells. HEK293 and Huh7 cells were plated in complete media containing glutamine for 24 h. One culture was collected for western blotting 24 h later (T0). The remainder of the cultures were washed with DPBS and fresh glutamine-depleted media with or without 0.8 mM NH_4_Cl was added. Cultures were collected at different time points and protein was extracted for western blotting. Protein abundance of phosphorylated S6K (T389) relative to total S6K protein levels (**A**,**C**) and phosphorylated ACC (S79) relative to total ACC protein levels (**B**,**D**) at 15 min, 30 min, 1 h, 2 h, 4 h and 6 h post-incubation was quantified using western blot. Blots shown were representative from 3 independent experiments. *p ≤ 0.05, **p ≤ 0.01, ***p ≤ 0.001. Full length blots are presented in Supplementary information Fig. 4.

**Figure 6:**
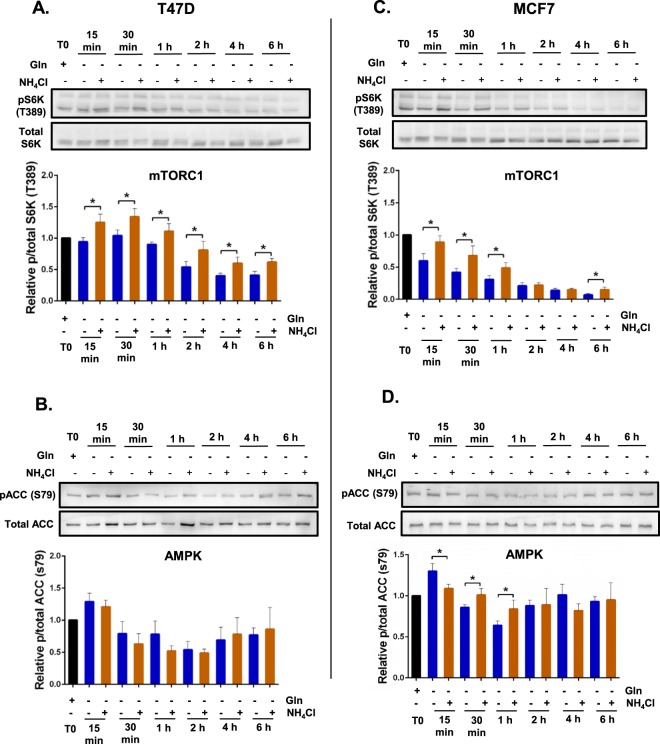
Acute glutamine depletion and ammonia lead to dynamic changes in mTORC1 and AMPK signalling in T47D and MCF7 cells. T47D and MCF7 cells were plated in complete media containing glutamine for 24 h. One culture was collected for western blotting 24 h later (T0). The remainder of the cultures were washed with DPBS and fresh glutamine-depleted media with or without 0.8 mM NH_4_Cl was added. Cultures were collected at different time points and protein was extracted for western blotting. Protein abundance of phosphorylated S6K (T389) relative to total S6K protein levels (**A**,**C**) and phosphorylated ACC (S79) relative to total ACC protein levels (**B**,**D**) at 15 min, 30 min, 1 h, 2 h, 4 h and 6 h post-incubation was quantified using western blot. *p ≤ 0.05, **p ≤ 0.01, ***p ≤ 0.001. Full length blots are presented in Supplementary information Fig. 4.

The AMPK and mTORC1 signalling responses are sensitive to nutrient availability and can be dynamic. Indeed, when we looked at the response over a six-hour period, the effect of NH_4_Cl supplementation (−gln + NH_4_Cl) compared to glutamine depletion alone (−gln) was indeed dynamic but varied between different cell lines (Figs 5 and 6). In all four cell lines glutamine depletion (−gln) decreased mTORC1 activity over the six hour period. NH_4_Cl supplementation (−gln + NH_4_Cl) reduced this starvation-induced inhibition of mTORC1 activity in Huh7, MCF7 and T47D (Figs 5 and 6). In contrast, in HEK293 cells, there was no significant effect of NH_4_Cl supplementation on mTORC1 activity (Fig. 5A).

Glutamine depletion (−gln) promoted a rapid increase in AMPK activity (Fig. 4B). However, this activation was transient as AMPK activity dropped in all four cell lines at later time points (Figs 5 and 6). Additional NH_4_Cl supplementation (−gln + NH_4_Cl) to glutamine-depleted culture reduced AMPK activity in HEK293, Huh7 and MCF7 cells at 15 min (Fig. 4B). However, interestingly, the effects of NH_4_Cl supplementation on AMPK activity were reversed in Huh7 and MCF7 cells at 30 min as AMPK activity increased compared to glutamine-depleted cells (Figs 5 and 6).Thus, the effect of NH_4_Cl on AMPK activity in a glutamine-depleted environment is very dynamic and cell line-dependent. These data also suggest that over a six-hour period, there is an effect of ammonia on mTORC1 activation that is independent of AMPK activity.

Considering HEK293 and Huh7 cells can both grow in glutamine depleted environment without an alternative nitrogen source, while both T47D and MCF7 cells showed limited ability to grow without glutamine, we chose to use one cell line from each group: HEK293 and T47D cells for further studies.

**Figure 7:**
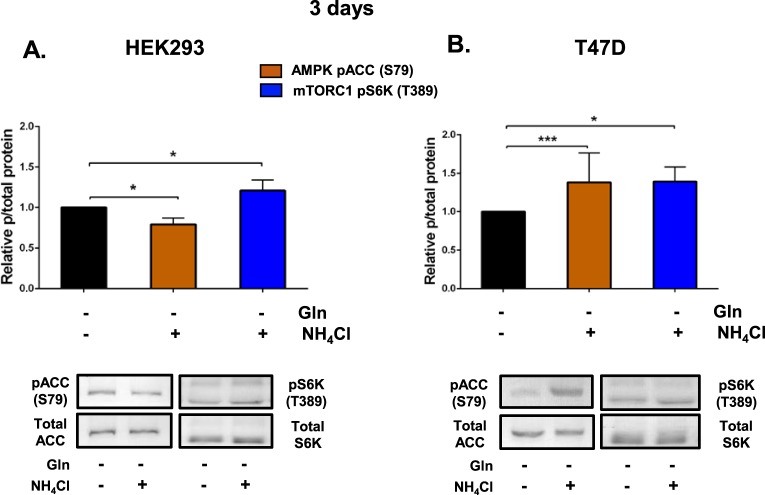
Chronic glutamine depletion and ammonia regulate AMPK and mTORC1 signalling in HEK293 and T47D cells. HEK293 and T47D cells were plated in complete medium containing glutamine for 24 h. Cultures were washed with DPBS and fresh glutamine-depleted media with or without NH_4_Cl was added. Cultures were collected 3 days later and protein was extracted for western blotting. Protein abundance of phosphorylated ACC (S79) or S6K (T389) relative to total ACC or S6K in HEK293 (**A**) and T47D (**B**) cells 3 days post-incubation was quantified using western blot. Blots shown were representative from 4 independent experiments. *p ≤ 0.05, **p ≤ 0.01, ***p ≤ 0.001. Full length blots are presented in Supplementary information Fig. 4.

We next assessed the effect of NH_4_Cl supplementation on AMPK and mTORC1 activity after 3 days. In HEK293 cells, NH_4_Cl slightly decreased the activity of AMPK, whilst mTORC1 activity was increased (Fig. 7A). In T47D cells, however, NH_4_Cl increased AMPK activity, while mTORC1 activity was also higher (Fig. 7B), which again suggest that there was an uncoupling between AMPK and mTORC1 activity. However, the persistent increase in mTORC1 activity in the NH_4_Cl supplementation group across the acute (15 min–6 h) and chronic (3 day) treatment periods in T47D (Figs 6A and 7B) and over 3 days in HEK293 cells (Fig. 7A) is consistent with the increased cell proliferation in the NH_4_Cl-supplemented cultures compared to glutamine depletion alone (Figs 1 and 3).

### AMPK activation partially reduced mTORC1 signalling and proliferation

**Figure 8:**
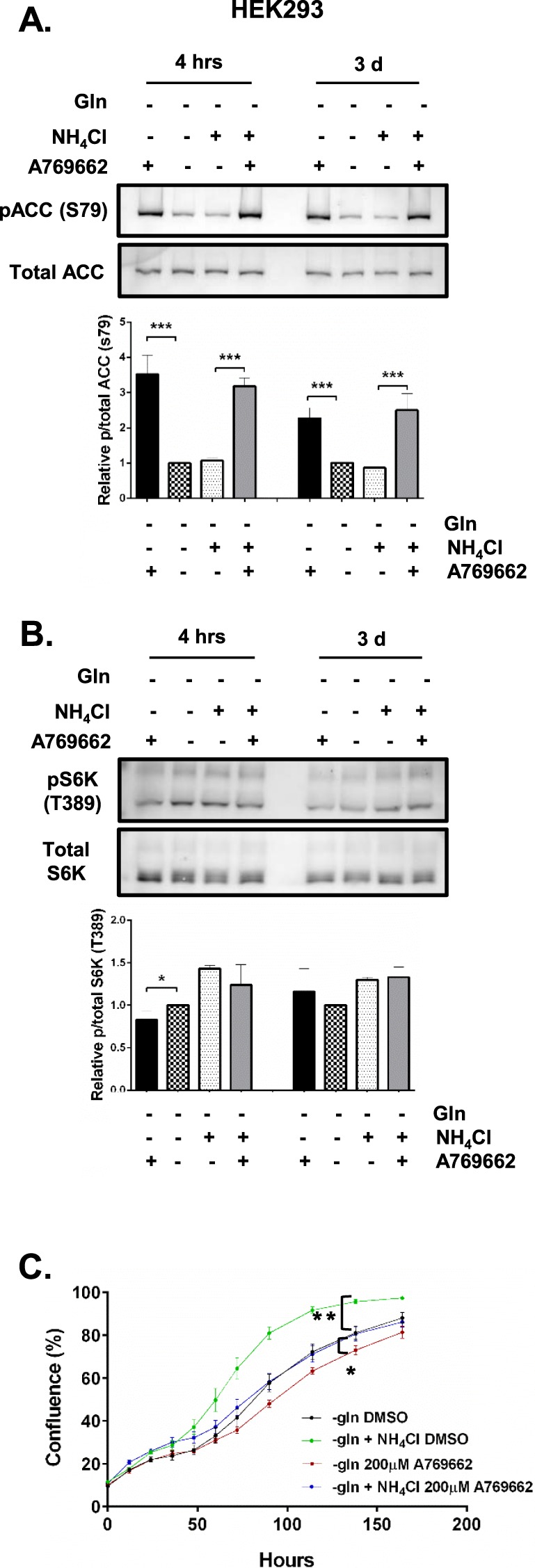
Effects of AMPK activation using A769662 on mTORC1 signalling and proliferation in HEK293 cells. HEK293 cells were plated in full media containing glutamine for 24 h. Cultures were washed with DPBS and fresh glutamine-depleted media with or without NH_4_Cl or A769662 (200 µM) was added. Cells cultured in glutamine-depleted media with or without NH_4_Cl or DMSO served as vehicle controls. Cultures were collected at 4 h and 3 days post-incubation for protein extraction or subjected to live imaging using IncuCyte FLR assay (n = 3) to measure cell proliferation rate over 7 days (**C**). Protein abundance of phosphorylated ACC (S79) (**A**) or S6K (T389) (**B**) relative to total ACC or S6K was quantified using western blot. Blots shown were representative from 3 independent experiments. *p ≤ 0.05, **p ≤ 0.01, ***p ≤ 0.001. Full length blots are presented in Supplementary information Fig. 4.

A769662 is a specific activator of AMPK while metformin is an anti-diabetic drug that is also well known to activate AMPK^17^. We utilised these agents to study the effect of AMPK activation (ACC phosphorylation) on mTORC1 signalling (S6K phosphorylation) and cell proliferation. A769662 (200 µM) effectively increased AMPK activity at 4 h and this response persisted up to 3 days in HEK293 cells cultured in glutamine depleted environment with or without NH_4_Cl (Fig. 8A). mTORC1 activity was lower at 4 h following A769662 treatment in cells cultured in glutamine-depleted media without NH_4_Cl, but was not affected in glutamine-depleted cells supplemented with NH_4_Cl (Fig. 8B). At day 3, mTORC1 activity was not changed following A769662 treatment in cells cultured in glutamine-depleted media with or without NH_4_Cl. This data again suggests that AMPK and mTORC1 signalling are not tightly coupled under these growth conditions. Interestingly, despite limited impact on mTORC1 activity, AMPK activation reduced cell growth of glutamine-depleted cultures supplemented with NH_4_Cl to the same slower rate as glutamine-depleted cells without NH_4_Cl (Fig. 8C). AMPK activation had a significantly smaller inhibitory effect on cell growth in glutamine-depleted cultures (Fig. 8C). Similarly, AMPK activation using metformin (5 mM) had limited impact on mTORC1 activity, however it reduced cell growth of glutamine-depleted cultures with or without NH_4_Cl to the same effect (Fig. S1A–C).

In T47D cells, the volume of DMSO in which the A769662 was diluted had an effect on cell proliferation, i.e., DMSO increased growth in the glutamine-depleted cultures (Fig. S2A). We tried to reduce the concentration of A769662 to 100 µM, thus reducing the amount of DMSO added to the culture, but DMSO still affected proliferation, such that it abolished the increase in cell growth in the NH_4_Cl supplemented cultures compared to glutamine depletion alone (Fig. S2B). Nevertheless, activation of AMPK using A769662 did decrease T47D proliferation in the glutamine-depleted cultures with or without NH_4_Cl compared to the corresponding DMSO controls (albeit not significant in the NH_4_Cl supplemented group (Fig. S2B). We also repeated this experiment using metformin (5 mM) as metformin was diluted in water, and we obtained similar results as in HEK293 (Fig. S2C–E).

Together these results demonstrate that in glutamine-depleted cells AMPK and mTORC1 signalling are not tightly coupled and that increased AMPK activity can reduce cell proliferation without having an impact on mTORC1 activity.

### GDH inhibition activates AMPK, reduced mTORC1 activity and cell proliferation

**Figure 9:**
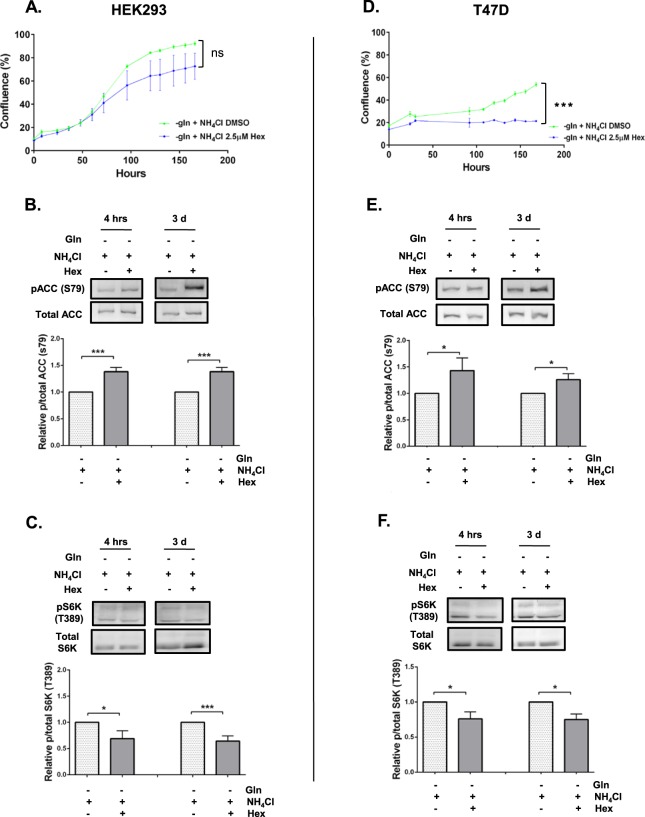
Effects of GDH inhibition on AMPK and mTORC1 activity and proliferation in HEK293 and T47D cells. HEK293 and T47D cells were plated in full media containing glutamine for 24 h. Cultures were washed with DPBS and fresh glutamine-depleted media with NH_4_Cl with or without hexachlorophene (Hex – 2.5 µM) was added. Cells cultured in glutamine-depleted media with or without NH_4_Cl or DMSO served as vehicle controls. Cultures were collected at 4 h and 3 days post-incubation for protein extraction or subjected to live imaging using IncuCyte FLR assay (n = 3) to measure cell proliferation rate over 7 days (**A**,**D**). Protein abundance of phosphorylated ACC (S79) (**B**,**E**) or S6K (T389) (**C**,**F**) relative to total ACC or S6K was quantified using western blot. Blots shown were representative from 3 independent experiments. *p ≤ 0.05, **p ≤ 0.01, ***p ≤ 0.001. Full length blots are presented in Supplementary information Fig. 4.

Previous studies have shown that GDH is the key enzyme in facilitating the ability of T47D and MCF7 cells to utilise NH_4_Cl as alternative nitrogen source to support proliferation^7,9^. In this study, we used a GDH inhibitor, hexachlorophene (Hex)^18^ and confirmed that GDH is critical in the utilisation of NH_4_Cl as an alternative nitrogen source to support proliferation in HEK293 and T47D cells (Fig. 9). At the lower concentration (2.5 µM), GDH inhibition partially reduced proliferation in HEK293 cells (Fig. 9A), while it abolished proliferation in T47D cells (Fig. 9D). Higher concentrations of GDH inhibitor (5 µM), however, also abolished proliferation of HEK293 cells (Fig. S3). Interestingly, GDH inhibition increased AMPK activity and reduced mTORC1 activity in HEK293 (Fig. 9B,C) and T47D (Fig. 9E,F) cells, which may in part explain the loss of the cell’s ability to proliferate when GDH activity is lost (Fig. 9A,D).

### Ulk1 inhibition reduced cell proliferation

**Figure 10:**
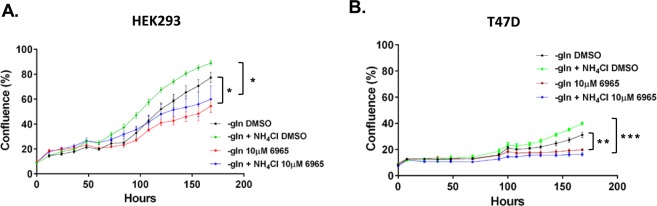
Effect of Ulk1 inhibition on proliferation in HEK293 and T47D cells. HEK293 and T47D cells were plated in full media containing glutamine for 24 h. Cultures were washed with DPBS and fresh glutamine-depleted media with or without NH_4_Cl or SBI0206965 (6965–10 µM) was added. Cells cultured in glutamine-depleted media with or without NH_4_Cl or DMSO served as vehicle controls. Cultures were subjected to live imaging using Incucyte FLR assay (n = 3) to measure cell proliferation rate over 7 days (**A**,**B**). *p ≤ 0.05, **p ≤ 0.01, ***p ≤ 0.001.

The ability of cells to survive without glutamine, may be due to the induction of autophagy. The serine/threonine protein kinase, Ulk1, is a key inducer of autophagy, which can be inhibited by a small molecule SBI0206965^19^. Treatment of HEK293 and T47D cells with this inhibitor decreased cell proliferation in glutamine-depleted cultures whether or not it was supplemented with NH_4_Cl (Fig. 10A,B).

## Discussion

Ammonia is a common metabolic by-product mainly produced in muscles during exercise and by proliferating cells^20,21^. Some of the ammonia produced in contracting muscle can be taken back up by the muscle cells and recycled to produce amino acids, particularly glutamine^20^. This is consistent with studies which showed that ^15^N labelled NH_4_Cl was incorporated into newly synthesised proteins in children and adults^22–24^. Interestingly, this incorporation of NH_4_Cl was more efficient in undernourished individuals^23^.

During cell proliferation, glutamine and glucose are the major nutrient inputs and one of the main by-products is ammonia^25^. In proliferating glioblastoma, about half of the nitrogen from glutamine metabolism is secreted from the cells into the extracellular space^26^. This may result in ammonia accumulation in the extracellular space, resulting in a higher level of ammonia in the tumor microenvironment compared to the circulation in healthy individuals (up to 50 µM)^14^. In individuals with hyperammonemia, circulating ammonia levels can reach up to 1 mM^14^. Surprisingly, NH_4_Cl at up to 50 mM was not toxic to breast cancer cell lines, T47D and MCF7. This is a concentration that is much higher than is considered toxic to primary human astrocytes (5 mM)^7^. Thus, in this and other studies, ~0.8 mM NH_4_Cl is considered non-toxic and optimum for alternative nitrogen source to support proliferation, as well as being a concentration relevant to the tumor microenvironment^7,10,12^.

In this study, as was reported before^7^, we show that not all cells can utilise ammonia (Fig. 1A). Interestingly, 0.8 mM NH_4_Cl is toxic to human prostate adenocarcinoma cells, LNCAP, and the human mammary cell line, MCF10A (Fig. 1B). This is somewhat surprising considering primary human astrocytes can tolerate up to 5 mM^7^. One explanation is that in LNCAP and MCF10A cells ammonia may lead to ER-stress induced apoptosis^27,28^. Higher concentrations of NH_4_Cl (4 mM) were also shown to stimulate autophagy^27,29^. Autophagy is a well-known cell survival mechanism and it has also been linked to cancer progression^30,31^. On the other hand, high levels of autophagy can also lead to autophagy-induced apoptosis^30,32^. Thus, the cell’s ability to tolerate or utilise ammonia for proliferation may depend on the cell’s ability to regulate autophagy.

We have also found that consistent with previous studies^7,9,11^, T47D and MCF7 cells can utilise NH_4_Cl to support proliferation (Fig. 1C). Interestingly, the human hepatocellular carcinoma, Huh7, and human embryonic kidney, HEK293 cells were able to not only utilise NH_4_Cl to support proliferation, but also to proliferate robustly in a glutamine-depleted environment without supplementation with ammonium chloride (Fig. 1D). Similarly, we found that over 7 days, T47D and MCF7 cells can also grow without glutamine, albeit to a much lesser extent (Fig. 3C,D). Thus, different cells have distinct tolerances towards ammonia and also perhaps a varying range of ‘optimum’ concentrations that can be utilised to support proliferation. Although glutamine is a non-essential amino acid, it is crucial to support proliferation^33^. Therefore, it is surprising that these cells, particularly Huh7 and HEK293, can proliferate robustly in the absence of glutamine. Again, one possibility points towards the cell’s ability to induce autophagy and thus support proliferation. Our data showed that when we incubated HEK293 and T47D cells with a well-known inhibitor of Ulk1 (SBI0206965)^19^, which is a key inducer of autophagy, cell proliferation was decreased in glutamine-depleted cultures whether or not it was supplemented with NH_4_Cl (Fig. 10A,B). This data provides evidence that perhaps induction of autophagy is important for cell survival and proliferation in a glutamine-depleted environment.

Glutamine synthetase (GS) and glutamate dehydrogense (GDH) are key enzymes in ammonia utilisation^6^, although recent studies showed that it is the GDH not GS enzymes that plays a key role in cell’s ability to utilise ammonia as alternative nitrogen source to support proliferation^7,9^. In this study we showed that the protein levels of GS or GDH do not correlate with the ability of cells to survive or utilise ammonia to support proliferation in glutamine depleted environment (Fig. 2), although GS levels were generally higher in cells that can utilise ammonia to support proliferation (T47D, HEK293 and Huh7), with the exception of MCF7. There were differences in GDH isoform expression, with MCF10A cells expressing high levels of the GDH isoform 1 and all other cells equally expressing lower levels. In contrast, MCF10A cells expressed the lowest levels of GDH isoform 2 compared to other cells. MCF10A cells cultures when supplemented with ammonia showed cell death (Figs 1B and 2B). Furthermore, the protein level of GDH isoform 3 was highest in Huh7 compared to all other cells tested (Fig. 2B). Huh7 showed the most robust growth in a glutamine depleted environment and this was further increased in the presence of ammonia (Figs 1D and 3B). These findings highlight the heterogeneity in GS and GDH gene expression between cell lines or cancer cells subtypes. Furthermore, different isoforms of GDH may play different roles in ammonia utilisation.

We hypothesised that only cancer cells have the ability to utilise ammonia as an alternative nitrogen source. However, here we found that the non-cancerous cell line HEK293 can utilise ammonia as alternative nitrogen source, while not all cancer cells have this ability. HEK293 cells constitutively express the Ad5 E1A/E1B protein which results in deregulation of one of the major tumor suppressors, p53, and it displays chromosomal instability^34^. Although these characteristics may explain why HEK293 can behave like a cancer cell, deregulation of the p53 pathway does not explain the ability of HEK293, Huh7, T47D and MCF7 to utilise ammonia as alternative nitrogen source, as Huh7 and T47D harbour p53 mutations resulting in a high level of p53 protein, while MCF7 cells does not harbour p53 mutations^35,36^ (Table 1). We also considered other common mutations in cancer namely KRAS, PTEN and PI3K. Mutation status of these genes also does not explain the ability of cells to utilise ammonia (Table 1).

**Table 1:** Mutations of cancer cell lines listed in the Cataloque of Somatic Mutations in Cancer (COSMIC).

Cell lines	Total number of mutations	KRAS	PTEN	PI3K	p53
SH-SY5Y	1	x	x	x	x
HCT-116	1109	√	x	√	x
HT29	733	x	x	x	x
LNCAP	20	x	√	x	x
T47D	181	x	x	x	√
MCF7	248	x	x	x	x

AMPK and mTORC1 are well established regulators of cell growth and proliferation which are directly regulated by varying nutrient sources^1,13^. In yeast, nitrogen sensing is independent of carbon and amino acid sensing pathways. In *S*. *pombe*, nitrogen stress activates AMPK and inhibits TORC1 to promote mitotic onset^12^. It is important to note that this decrease in TORC1 activity and regulation of mitosis occurred independently of glucose availability. In hepatocellular carcinoma cells, Hep3B glutamine starvation also reduced mTORC1 activity^12^. In this study we show that glutamine depletion also resulted in decreased mTORC1 activity in T47D, MCF7, HEK293 and Huh7 cells over 6 h (Figs 5A,C and 6A,C), in high levels of glucose. Furthermore, consistent with our previous findings in the Hep3B cells, NH_4_Cl supplementation re-established mTORC1 signalling to a varying degrees in T47D, MCF7 and Huh7 cells, but not HEK293 after 6 hr. However, NH_4_Cl supplementation supported cell proliferation of all four cell lines when starved of glutamine (Fig. 3A–D) and consistently, in HEK293 and T47D NH_4_Cl supplementation enhanced mTORC1 at day 3 (Fig. 7).

Surprisingly we observed an increase in AMPK activity in response to acute (15 min) glutamine depletion in all four cell lines. Although AMPK is known to be activated in response to nutrient deprivation, particularly glucose, this is the first report, which showed that AMPK was also upregulated in response to deprivation of a single amino acid (Fig. 3B). Furthermore our data revealed that NH_4_Cl resulted in a dynamic alteration of AMPK activity that is cell dependent (Figs 5B,D and 6B,D). At 15 min post-incubation, NH_4_Cl supplementation decreased AMPK activity (Fig. 3). However, in Huh7 (30 min to 2 h) and MCF7 (30 min to 1 h), NH_4_Cl supplementation increased AMPK activity compared to glutamine-starved controls (Fig. 5D). This is consistent with one study, albeit at much higher NH_4_Cl concentration (4 mM), which showed that NH_4_Cl increased AMPK activity and a concomitant induction of autophagy^27^

AMPK is a well-established inhibitor of mTORC1, which in turn promotes proliferation^1,13^. Thus, we assessed the effects of activating AMPK on mTORC1 activity and the ability of cells to utilise NH_4_Cl as an alternative nitrogen source to support cell proliferation when starved of glutamine. The specific AMPK activator, A769662, and non-specific metformin increased AMPK activity as expected. Although this had a limited impact on mTORC1, proliferation was decreased (Figs 8A–C, S1A–C and S2B–E). This suggests that under these conditions when cell are deprived of glutamine AMPK has limited impact on mTORC1 signalling. Presumably AMPK control of mitosis and cell division accounts for mTORC1 independent inhibition of cell proliferation^37^.

Consistent with previous studies, we have confirmed that GDH is essential in facilitating cell’s ability to utilise NH_4_Cl as an alternative nitrogen source^7,9^. Here, we showed that GDH inhibition leads to increased AMPK activation and decreased mTORC1 activity, suggesting that GDH, AMPK and mTORC1 are closely linked and are critical in the ability to utilise NH_4_Cl as an alternative nitrogen source. Interestingly, as different cells have varying ability to utilise or tolerate NH_4_Cl, we found that HEK293 cells were more resistant to GDH inhibition than T47D (Figs 9C and S3). This regulation of AMPK by GDH is supported by a previous finding in endothelial cells whereby inhibition of GDH reduced inflammation, in which the effect was abolished when AMPK was inhibited^38^. Similarly, GDH inhibition reduced low glucose-induced activation of AMPK in myotubes and β-islet cells^39^. Furthermore, mTOR inhibition resulted in GDH inhibition leading to reduced glutamine-induced proliferation in ovarian cancer cells^40^.

In summary, we have confirmed that ammonia is not simply a metabolic waste product but can help support cell proliferation. This study extends previous findings and shows that not all cells can utilise ammonia as alternative nitrogen source. Indeed, different cells have different tolerance or ability to utilise ammonia to support proliferation. We have shown that GDH, AMPK and mTORC1 are linked and that AMPK and mTORC1 are dynamically regulated to support proliferation in response to glutamine depletion and in NH_4_Cl utilisation as an alternative nitrogen source (summarised in Table 2). Despite the common understanding that glutamine is crucial for proliferation, we found that Huh7, HEK293 and, to a much lesser extent, breast cancer cells (T47D and MCF7) can proliferate in glutamine-depleted environment. Tumor cells grow under relatively nutrient deprived environment, yet they can proliferate at a much higher rate than normal cells. Ammonia is a common by-product of proliferating cells. Thus, this and other studies have provided evidence that one strategy of the tumor to be able to grow rapidly in a nutrient deprived environment may be the ability to recycle the metabolic by-product ammonia to support proliferation.

**Table 2:** Effects of glutamine starvation and ammonia supplementation on AMPK and mTORC1 signalling and cell growth. Upward and downward arrows indicate an increase or decrease in kinase activity or increase in cell growth respectively. Two-way arrows indicate no change.

Cell line	Gln starvation (compared to + Gln)		NH_4_Cl supplementation (compared to −Gln)						
	Acute (15 mins)		Acute (15 mins)		Acute (6 hrs)		Day 3		
	AMPK activation	mTORC1 activation	AMPK activation	mTORC1 activation	AMPK activation	mTORC1 activation	Growth	AMPK activation	mTORC1 activation
HEK293	↑	↓	↓	↔	↔	↔	↑	↓	↑
Huh7	↑	↔	↔	↑	↔	↑	↑	—	—
T47D	↑	↓	↓	↑	↔	↑	↑	↑	↑
MCF7	↑	↓	↓	↑	↔	↑	↑	—	—

## Material and Methods

### Cell culture

Dulbecco’s Modified Eagle’s Medium (DMEM), fetal bovine serum (FBS), dialysed FBS, penicillin/streptomycin, glutamine, metformin hydrochloride and hexachlorophene (Hex) were purchased from Sigma Aldrich (St. Louis, MO). A769662 was purchased from Abcam (Cambridge, UK). SBI0206965 (6965) was purchased from Cayman chemical (Ann Arbor, MI).

HEK293, T47D, Huh7, MCF7, A549, HACAT, MCF10A, LNCAP, HT29, HCT116 and SH-SY5Y mammalian cell lines were cultured in DMEM with high glucose (4.5 g/L) supplemented with 10% FBS, 1% penicillin/streptomycin and 8 mM glutamine. For growth assays and cell lysates collected for western blot, cells were washed with Dulbecco’s phosphate buffered saline (DPBS) prior to culture with normal medium (Control (+gln); DMEM high glucose, 10% dialysed FBS, 1% penicillin/streptomycin and 8 mM glutamine) or glutamine depleted media with (−gln + NH_4_Cl) or without 0.8 mM ammonium chloride (−gln). Additionally, cells cultured in glutamine depleted medium with or without ammonium chloride were treated with or without the AMPK activator, A769662 (100 µM or 200 µM) or metformin (5 mM), GDH inhibitor, hex (2.5 µM or 5 µM) or the Ulk1 inhibitor, 6965 (10 µM). Control cells were cultured in glutamine depleted medium with or without ammonium chloride and dimethyl sulfoxide (DMSO) or water as vehicle control.

### Growth assays

Cell growth/density was quantified by fixing and staining the cells with 4% formaldehyde in DPBS with 0.5% crystal violet or measured by live imaging using the Incucyte FLR (Essen Bioscience, Ann Arbor, MI) assay. For the growth assay using crystal violet staining, cells were seeded at 2–5 × 10^4^ in 12 well plates (n = 6 per group). Cells in all groups were washed with DPBS 24 h after seeding. In the control group cells were immediately fixed after a DPBS wash. Cells in the positive control (+gln) and glutamine depleted with (−gln + NH_4_Cl) or without (−gln) ammonium chloride groups were cultured for 3 days before fixing and staining in 4% formaldehyde with 0.5% crystal violet. Excess stain was washed away with water. To measure the intracellular crystal violet staining, cells were bleached by incubation in 1% SDS in DPBS solution for 1 h and colorimetric (OD 540 nm) measurement was quantified using a spectrophotometer.

For the growth assay quantified using the Incucyte, HEK293, Huh7, MCF7 and T47D cells were seeded at 1–5 × 10^4^ in 12 well plates (n = 3 per group). Live cell density was measured over 7 days.

### Western Blot

HCT116, MCF10A, T47D, MCF7, HEK293 and Huh7 cells were seeded to 6 cm dishes before collection 24 hrs later at a cell confluence of 70% or below. HEK293, Huh7, MCF7 and T47D cells were seeded at 4–7 × 10^5^ in 35 mm dishes for the acute treatment (15 min to 6 h). HEK293 and T47D cells were plated at 2 × 10^5^ in 6 well plates for collection at day 3. Twenty four hours after seeding, cells were washed with DPBS and fresh glutamine depleted media with or without ammonium chloride and/or A769662, metformin or hex was added to the culture. At the designated time points, supernatant was collected, cells were washed with DPBS and the wash was combined with the supernatant. This was then centrifuged at 1000 g for 2 min to pellet the loosely adherent cells. Cells were scraped from the dish in 20% trichloroacetic acid (TCA) solution to precipitate the protein and the dish was washed with 5% TCA to collect the remainder of the protein in the dish. The 5% TCA wash was used to precipitate the protein in the pelleted loosely-adherent cells and combined to the precipitates in 20% TCA. Glass beads were added to the protein precipitates and lysed using Fast prep-24 homogeniser (MPBio, Santa Ana, CA) to break chromosomal DNA. Total protein was pelleted using centrifugation at 13,000 g for 3 min at 4 °C. SDS loading buffer, Tris pH 8 (to neutralise the samples) and 10 mM DTT were added to resuspend the protein pellet.

Proteins were separated by SDS polyacrylamide gels electrophoresis and transferred onto PVDF membrane (Milipore, Darmstadt, Germany). Membranes were incubated with primary antibodies to detect total ACC, phospho ACC (Ser79), total S6K or phospho S6K (Thr389) (1:1000, Cell Signaling, Danvers, MA), total GS (80636, 1:1000, Cell Signaling, Danvers, MA), total GDH (D9F7P, 1:1000, Cell Signaling, Danvers, MA) overnight at 4 °C. Membranes were then washed with 0.1% TBS tween and incubated with alkaline phosphatase conjugated secondary antibody for 1 h at room temperature. Membranes were then washed with 0.1% TBS tween and protein bands were developed using alkaline phosphatase buffer containing nitro blue tetrazolium chloride (NBT) and 5-bromo-4-chloro-3-indolyl-phosphate (BCIP) reagents (Sigma Aldrich). Density of protein bands were quantified using AlphaEaseFC 4.0 software. For western blot the relative signal density shown in the graphs were averaged density ± standard deviation from 3 independent experiments. Blots shown were representative from 3 independent experiments.

### Statistical analysis

Statistical significance was calculated using two-tailed student’s t-test or One-way ANOVA followed by Duncan Post-hoc to show significant difference between groups. P ≤ 0.05 was considered statistically significant. *p ≤ 0.05, **p ≤ 0.01, ***p ≤ 0.001.

## Supplementary information

Supplementary Figures 1–4.

## Data Availability

All data generated or analyzed during this study are included in this published article (and its Supplementary Information file).
