# Supplementary material for: The ability to utilise ammonia as nitrogen source is cell type specific and intricately linked to GDH, AMPK and mTORC1
Source: Sci Rep. 2019 Feb 6;9:1461. doi: 10.1038/s41598-018-37509-3 (PMC6365639; doi:10.1038/s41598-018-37509-3)

The ability to utilise ammonia as nitrogen source is cell type specific and intricately linked to GDH, AMPK and mTORC1. (max 20 words)

Shervi Lie[1], Tingting Wang[1], Briony Forbes[1], Christopher G. Proud[2] & *Janni Petersen[1,2,]

[1] Flinders Centre for Innovation in Cancer, College of Medicine and Public health, Flinders University, Adelaide, SA 5042, Australia.

[2] Nutrition and Metabolism, South Australia Health and Medical Research Institute, North Terrace, PO Box 11060, Adelaide, SA 5000, Australia.

*Corresponding author email: janni.petersen@flinders.edu.au

Supplementary figures

Supplementary Figure 1. Effects of AMPK activation using metformin on mTORC1 activity and proliferation in HEK293 cells

HEK293 cells were plated in full media containing glutamine for 24 h. Cultures were washed with DPBS and fresh glutamine-depleted media with or without NH_4Cl or metformin (5 mM) was added. Cells cultured in glutamine-depleted media with or without NH_4Cl or water served as vehicle controls. Cultures were collected at 4 h and 3 days post-incubation for protein extraction or subjected to live imaging using Incucyte FLR assay (n = 3) to measure cell proliferation rate over 7 days (C). Protein abundance of phosphorylated ACC (S79) (A) or S6K (T389) (B) relative to total ACC or S6K was quantified using western blot.

Relative signal density shown in the graphs were averaged density ± standard deviation from 2 independent experiments. Blots shown were representative from 2 independent experiments.

* $p \leq 0.05$, ** $p \leq 0.01$, *** $p \leq 0.001$.

Full length blots are presented in Supplementary information, Supplementary Figure 1.

Supplementary Figure 2. Effects of DMSO and AMPK activation on T47D cell proliferation

T47D cells were plated in full media containing glutamine for 24 h. Cultures were washed with DPBS and fresh glutamine-depleted media with or without NH_4Cl or DMSO or A769662 (100 µM) or metformin (5 mM) was added. Cultures were subjected to live imaging using Incucyte FLR assay (n = 3) to measure cell proliferation rate over 7 days (A, B & E) or collected at 4 h and 3 days post-incubation for protein extraction. Protein abundance of phosphorylated ACC (S79) (C) or S6K (T389) (D) relative to total ACC or S6K was quantified using western blot.

Relative signal density shown in the graphs were averaged density ± standard deviation from 2 independent experiments. Blots shown were representative from 2 independent experiments.

* $p \leq 0.05$, ** $p \leq 0.01$, *** $p \leq 0.001$.

Full length blots are presented in Supplementary information, Supplementary Figure 1.

Supplementary Figure 3. Effect of high dose of GDH inhibition on proliferation in HEK293 cells

HEK293 cells were plated in full media containing glutamine for 24 h. Cultures were washed with DPBS and fresh glutamine-depleted media with or without NH_4Cl or hexachlorophene (Hex – 5 μM) was added. Cells cultured in glutamine-depleted media with or without NH_4Cl or DMSO served as vehicle controls. Cultures were subjected to live imaging using Incucyte FLR assay (n = 3) to measure cell proliferation rate over 7 days.

* $p \leq 0.05$, ** $p \leq 0.01$, *** $p \leq 0.001$.

Supplementary Figure 4. Full length blots of all Western blots.

(A) Full length blots for figure 2. (B) Full length blots for figure 5. (C) Full length blots for figure 6. (D) Full length blots for figure 7. (E) Full length blots for figure 8. (F) Full length blots for figure 9. (G) Full length blots for figure S1 (H) Full length blots for figure S2

Supplementary Figure 1.

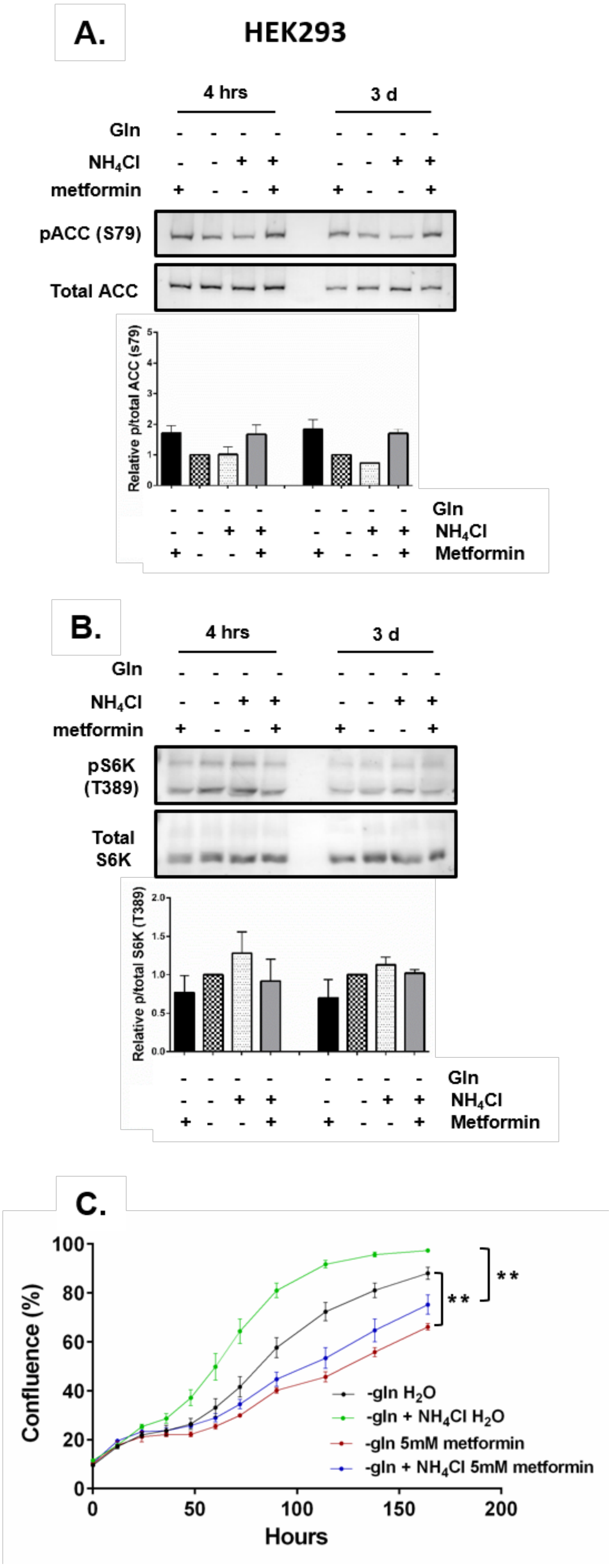

Supplementary Figure 2.

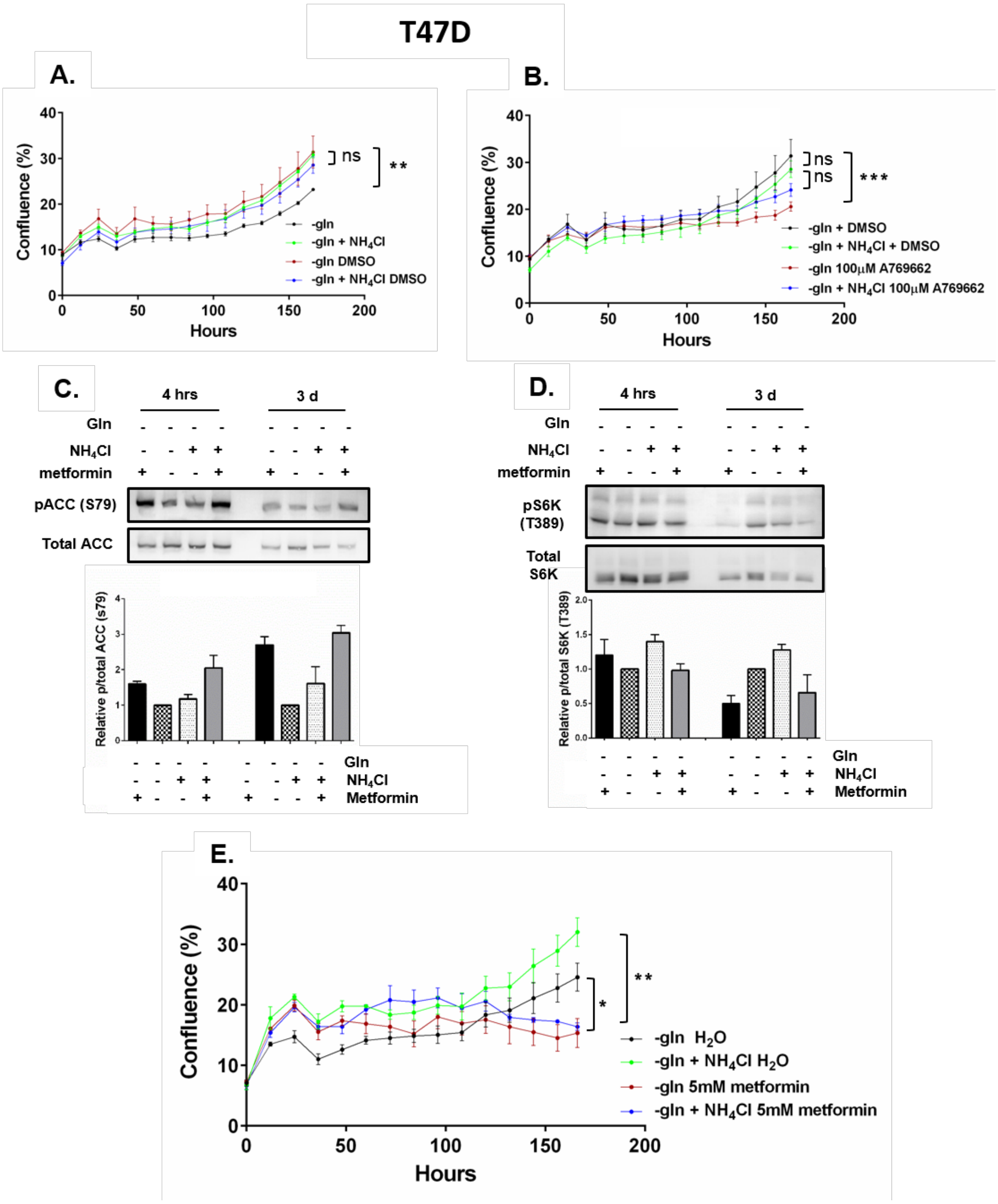

Supplementary Figure 3.

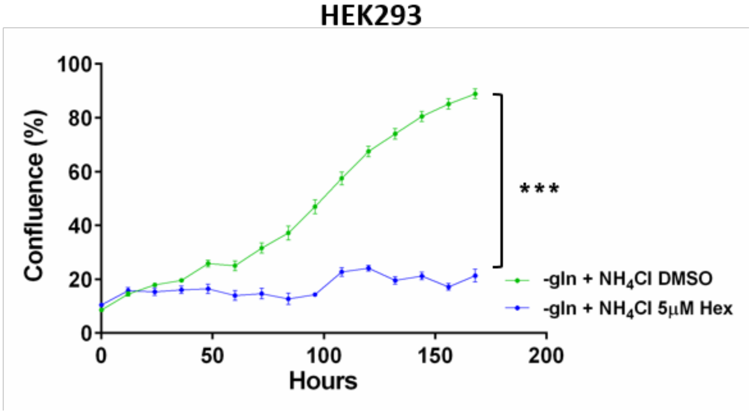

Supplementary Figure 4A

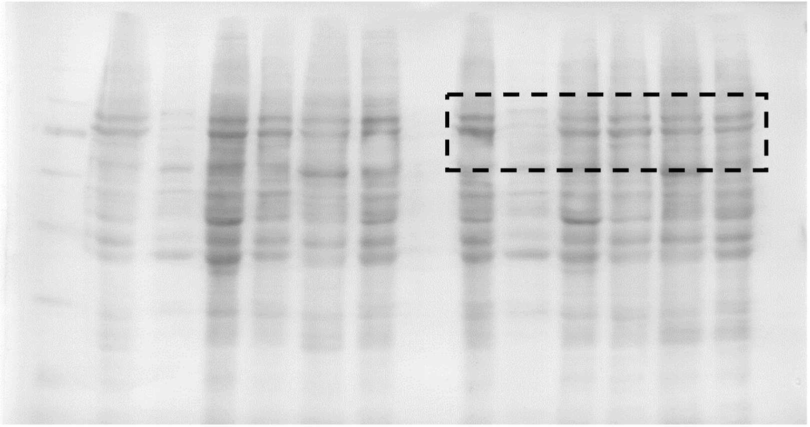

GS ponceau

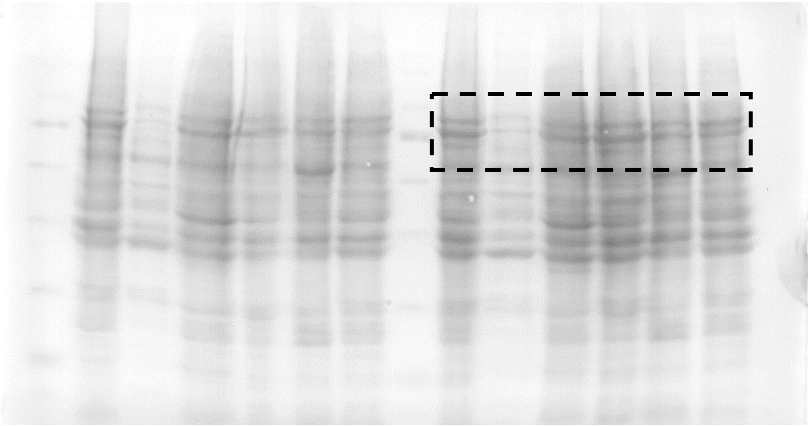

GDH ponceau

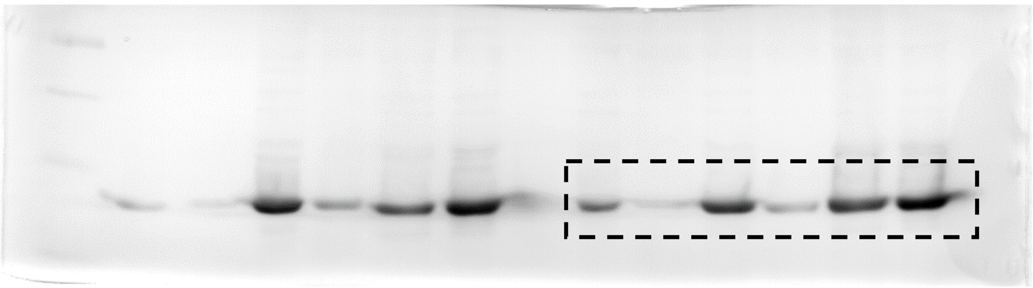

GS

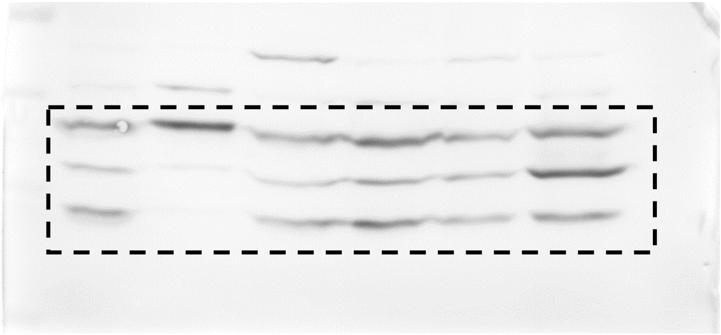

GDH

Supplementary Figure 4B

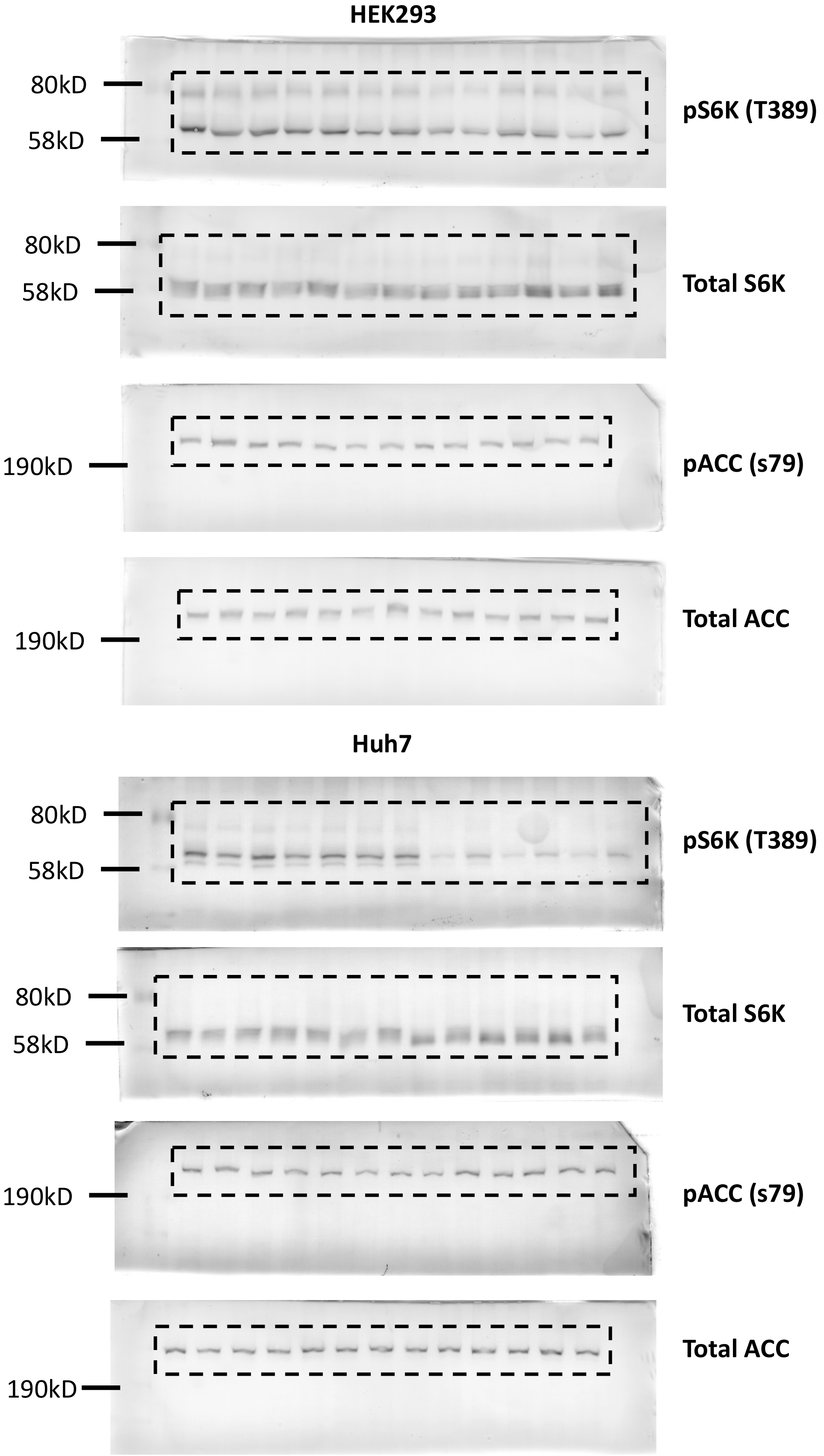

Supplementary Figure 4C

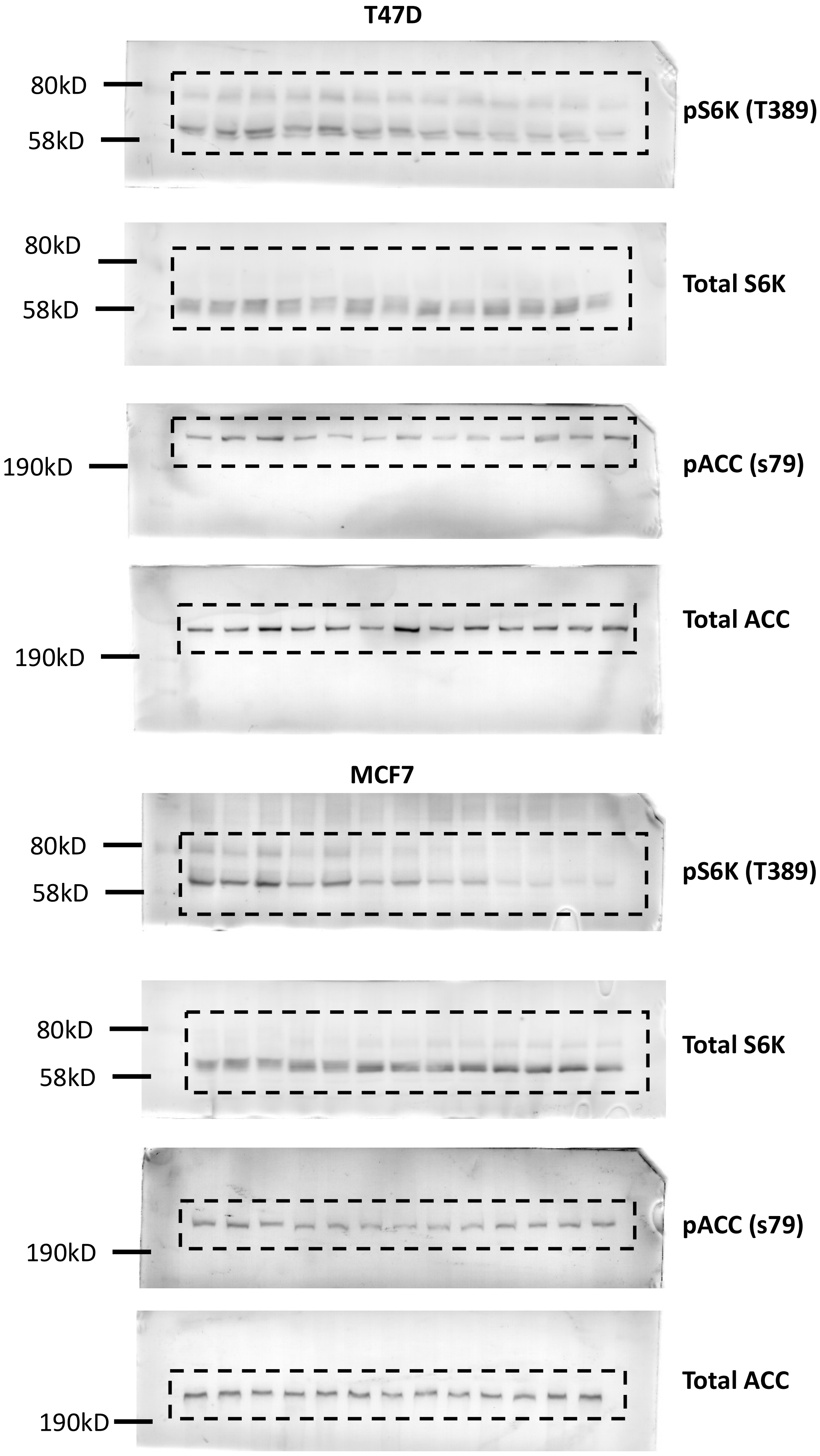

Supplementary Figure 4D

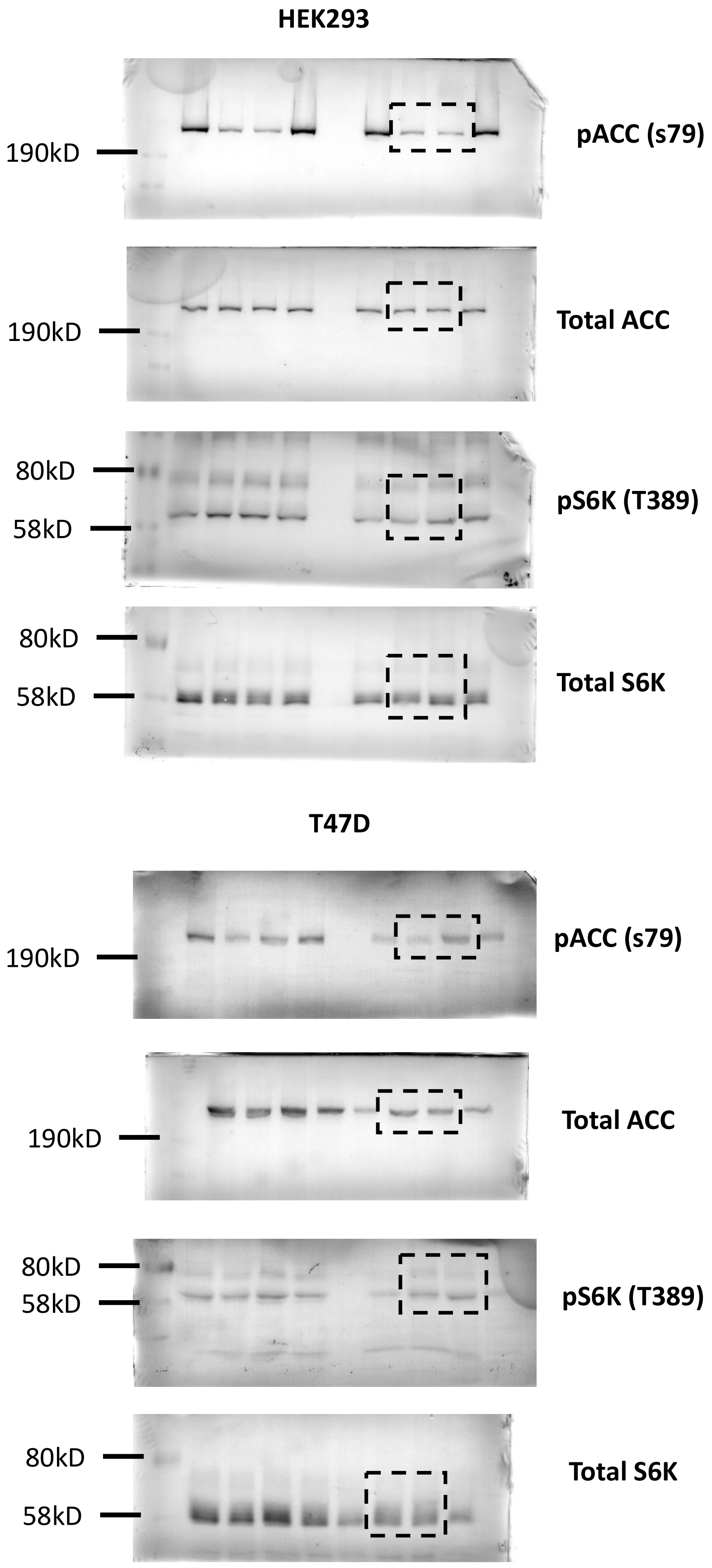

Supplementary Figure 4E

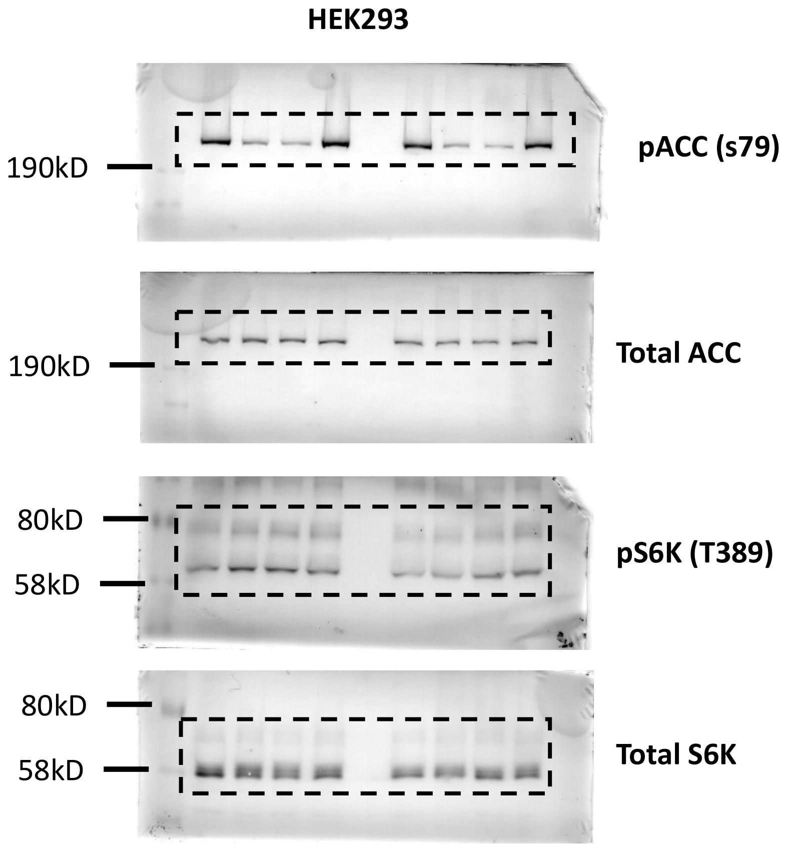

Supplementary Figure 4F

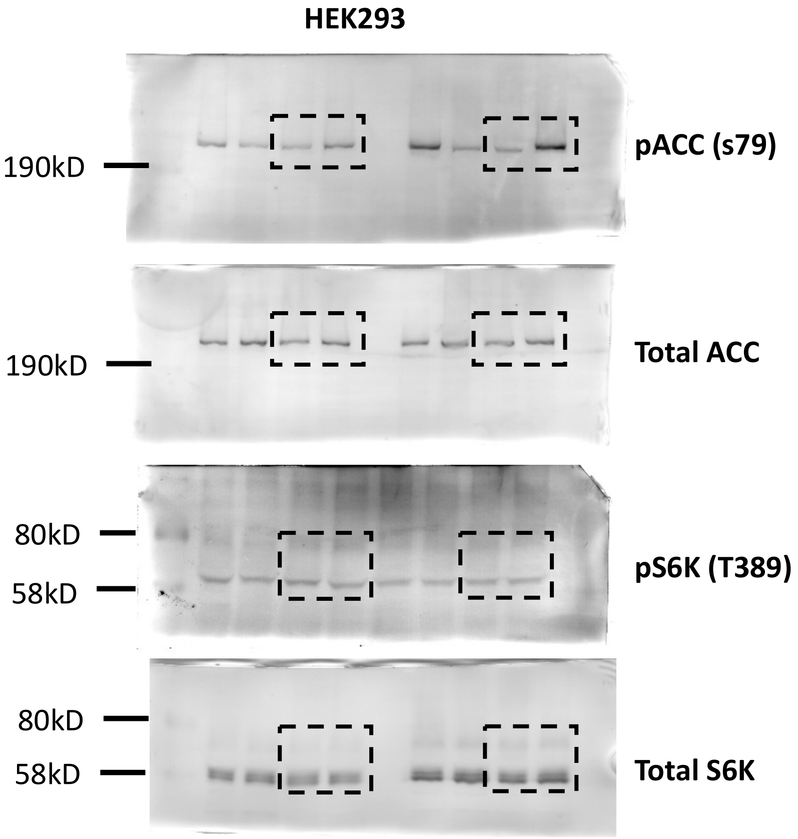

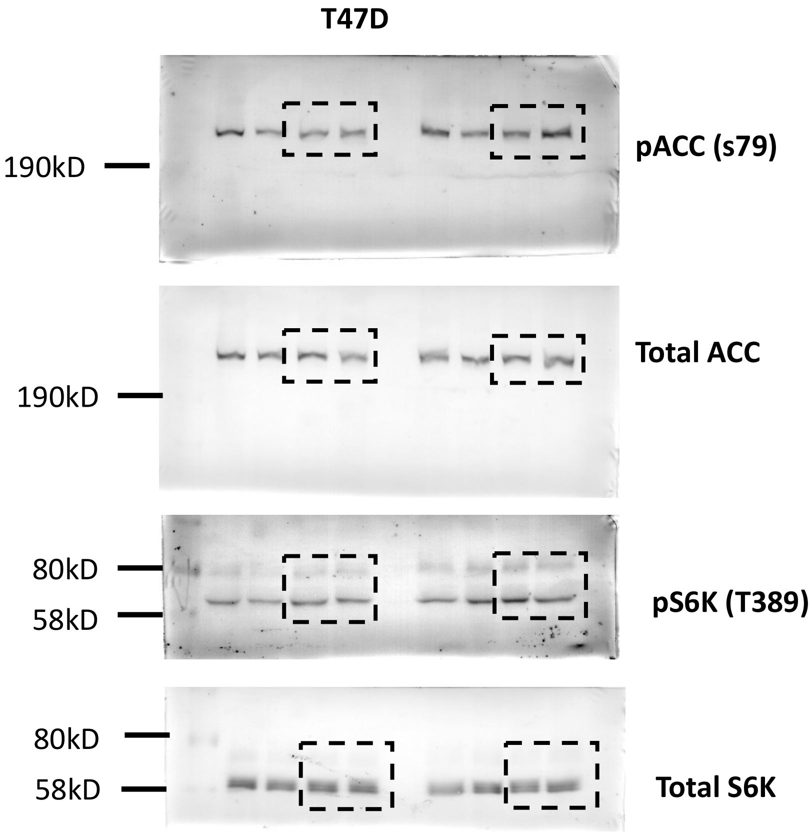

Supplementary Figure 4G

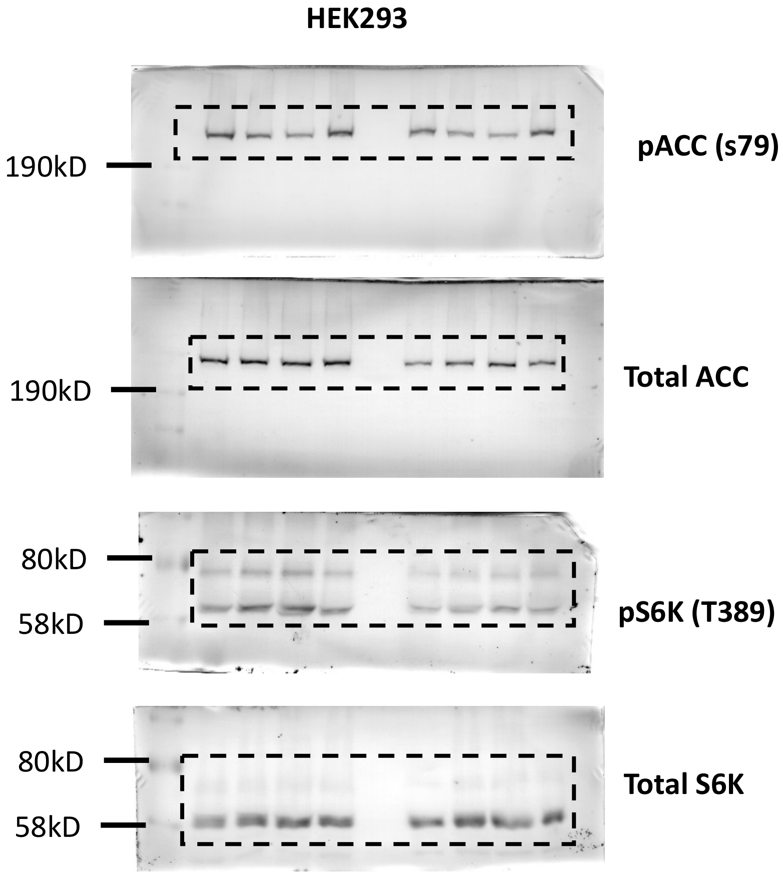

Supplementary Figure 4H

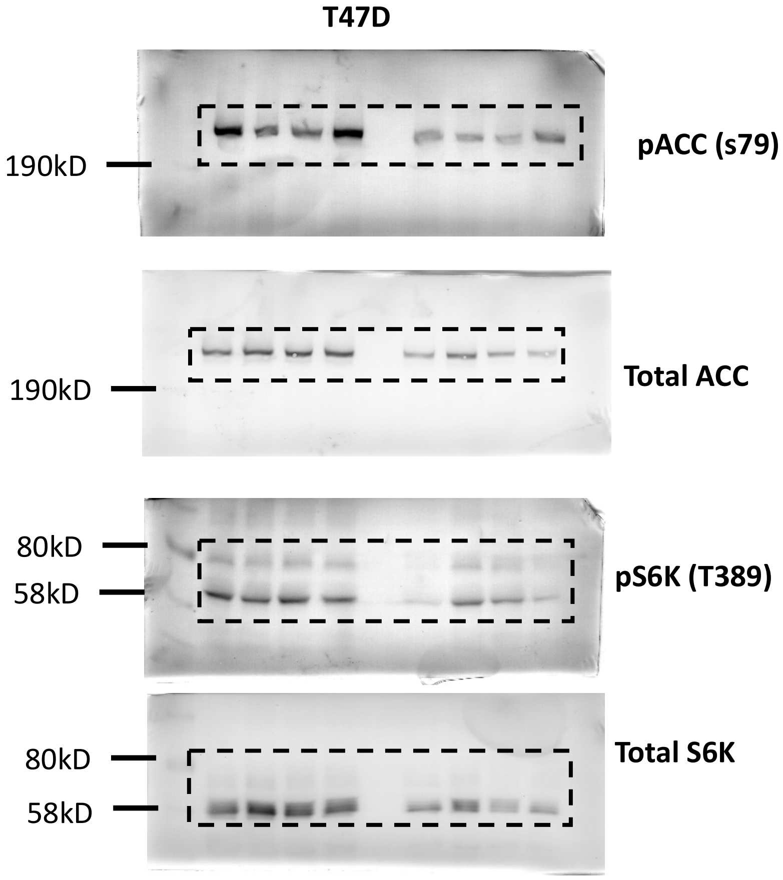

Supplement: Supplementary file 1 — Supplementary Figures 1–4. [file 41598_2018_37509_MOESM1_ESM.pdf]
